# Methuosis Inducer SGI‐1027 Cooperates with Everolimus to Promote Apoptosis and Pyroptosis by Triggering Lysosomal Membrane Permeability in Renal Cancer

**DOI:** 10.1002/advs.202404693

**Published:** 2024-08-09

**Authors:** Yu Luo, Bing Guan, Xiaoqi Deng, Peide Bai, Haichao Huang, Chaohao Miao, Anran Sun, Zhipeng Li, Dianqiang Yang, Xuegang Wang, Zhiqiang Shao, Yulong Wu, Jinchun Xing, Bin Chen, Tao Wang

**Affiliations:** ^1^ The Key Laboratory of Urinary Tract Tumors and Calculi Department of Urology The First Affiliated Hospital of Xiamen University School of Medicine Xiamen University Xiamen Fujian 361003 P. R. China; ^2^ Department of Urology The First Affiliated Hospital of Chongqing Medical University Yuzhong Chongqing 400016 P. R. China; ^3^ Department of Nephrology Zigong Fourth People's Hospital Zigong Sichuan 643000 P. R. China; ^4^ State Key Laboratory of Cellular Stress Biology School of Life Sciences Xiamen University Xiamen Fujian 361102 P. R. China; ^5^ Xiamen University Laboratory Animal Center Xiamen University Xiamen Fujian 361102 P. R. China; ^6^ Department of Urology The Fifth Hospital of Xiamen Xiamen Fujian 361101 P. R. China

**Keywords:** everolimus, lysosome, methuosis, pyroptosis, renal cell carcinoma, SGI‐1027

## Abstract

The mTOR inhibitor everolimus has been approved as a sequential or second‐line therapy for renal cell carcinoma (RCC). However, the development of drug resistance limits its clinical applications. This study aims to address the challenge of everolimus resistance and provide new insights into the treatment of advanced RCC. Here, the cytotoxicity of the DNA methyltransferase 1 (DNMT1) inhibitor SGI‐1027 in inducing cell vacuolation and methuosis is discovered and demonstrated for the first time. Additionally, SGI‐1027 exerts synergistic effects with everolimus, as their combination suppresses the growth, migration, and invasion of renal cancer cells. Mechanistically, apoptosis and GSDME‐dependent pyroptosis triggered by lysosomal membrane permeability (LMP) are observed. The upregulation of GSDME expression and increased lysosomal activity in renal cancer cells provide a therapeutic window for the combination of these two drugs to treat renal cancer. The combination treatment exhibits effective anti‐tumor activity and is well tolerated in a subcutaneous tumor model. Overall, this study validates and reveals the specific cytotoxicity property of SGI‐1027 and its potent synergistic effect with everolimus, offering new insights into advanced RCC therapy and everolimus‐resistance overcoming.

## Graphical abstract

Schematic model for cell death induced by SGI‐1027 in combination of everolimus suggested by the present study: SGI‐1027 induces vacuolation by macropinocytosis and inhibits the fusion of vacuoles and lysosomes. Everolimus enhances the process of macropinocytosis and cooperates with SGI‐1027 to induce lysosomal membrane permeability (LMP), subsequently leading to apoptosis and GSDME‐dependent pyroptosis.

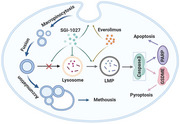

## 1 Introduction

Renal cell carcinoma (RCC) is one of the most common urological tumors with an increasing incidence.^[^ ^1^ ^]^ Despite the early detection of RCC achieved through widespread use of cross‐sectional abdominal imaging and improved patient access to surgery,^[^ ^2^, ^3^ ^]^ metastatic RCC and postoperative recurrence remain challenging in clinical.^[^ ^4^, ^5^ ^]^ Additionally, advanced RCC shows unsatisfactory response to radiotherapy, chemotherapy, and targeted therapy,^[^ ^6^, ^7^ ^]^ emphasizing the importance of exploring new therapeutic agents or treatment options. Everolimus has been approved by the Food and Drug Administration (FDA) for the treatment of advanced metastatic RCC.^[^ ^8^ ^]^ Phase III clinical trial has shown that everolimus improved the progression‐free survival (PFS) of patients with metastatic RCC.^[^ ^9^ ^]^ Despite this, the development of drug resistance significantly affects the clinical effectiveness of everolimus.^[^ ^10^, ^11^ ^]^ Currently, multiple mechanisms of everolimus resistance have been identified, such as the activation of the ERK/MAPK, PI3K/AKT pathways and autophagy activity in tumor cells,^[^ ^12^, ^13^ ^]^ which underscores the importance of multi‐target combination therapy for overcoming everolimus resistance. For example, everolimus combined with the ERK inhibitor SCH772984 and the autophagy inhibitor chloroquine have been reported to improve the therapeutic efficacy of everolimus in renal cancer cells in vitro and in vivo.^[^ ^14^ ^]^ However, most studies have been confined to the basic research stage, possibly due to the limited therapeutic efficacy of these combination therapy. Hence, it is imperative to identify the optimal drug combination with a robust synergistic effect and acceptable tolerance to overcome everolimus resistance, potentially yielding significant clinical benefits for patients with advanced RCC.

Most chemotherapy and targeted therapy agents exert their anti‐tumor effects by inducing apoptosis.^[^ ^15^, ^16^ ^]^ However, tumor cells can develop resistance to apoptosis by various mechanisms such as increasing drug efflux, enhancing DNA damage repair, or altering apoptosis pathways.^[^ ^17^, ^18^, ^19^ ^]^ Therefore, exploring alternative drugs targeting nonapoptotic cell death might be a potential way for antiapoptotic drug resistance. In recent years, methuosis has attracted attention as a potential alternative mechanism, given its peculiar form of non‐apoptotic cell death.^[^ ^20^ ^]^ Cells undergoing methuosis generate numerous cytoplasmic vacuoles through macropinocytosis.^[^ ^21^ ^]^ The vacuoles tend to accumulate, fuse, and expand because they neither recycle nor fuse with lysosomes.^[^ ^20^ ^]^ An increasing number of methuosis inducers have been reported in recent years. The chalcone‐related small molecule MIPP has been reported to induce methuosis in glioblastoma cells.^[^ ^22^ ^]^ The compound Epimedokoreanin C (EKC) isolated from a Chinese herb *Epimedium koreanum* has been reported to induce methuosis in lung cancer cells.^[^ ^23^ ^]^ The azaindole‐based compound 13 has been reported to induce methuosis in a variety of tumor cells.^[^ ^24^ ^]^ By contrast, pyroptosis is an intense programmed cell death mediated by the gasdermin protein family (GSDMs).^[^ ^25^ ^]^ Unlike apoptosis, pyroptosis is characterized by cell membrane breakdown and inflammatory cytokine release, activating the immune system and leading to inflammation.^[^ ^26^ ^]^ Pyroptosis has been considered as a new direction of chemoresistance and immunotherapy due to its natural proinflammatory ability and violent cellular response.^[^ ^27^ ^]^ Therefore, methuosis and pyroptosis could be the new directions for treatment of the anti‐apoptotic tumors.

In the present study, we discovered and demonstrated for the first time that SGI‐1027 induced cytoplasmic vacuolization and methuosis in renal cancer cells. In addition, SGI‐1027 cooperated with everolimus to induce apoptosis and GSDME‐dependent pyroptosis by triggering lysosomal membrane permeability (LMP). The increased expression of GSDME and lysosomal activity in renal cancer cells provided strong evidence for the combination of SGI‐1027 and everolimus in the treatment of advanced RCC. Generally, the unique pharmacological properties of SGI‐1027 offer new insights for RCC therapy and everolimus resistance.

## 2 Results

### 2.1 SGI‐1027 Induced Vacuoles via Macropinocytosis in Renal Cancer Cells

**Figure 1:**
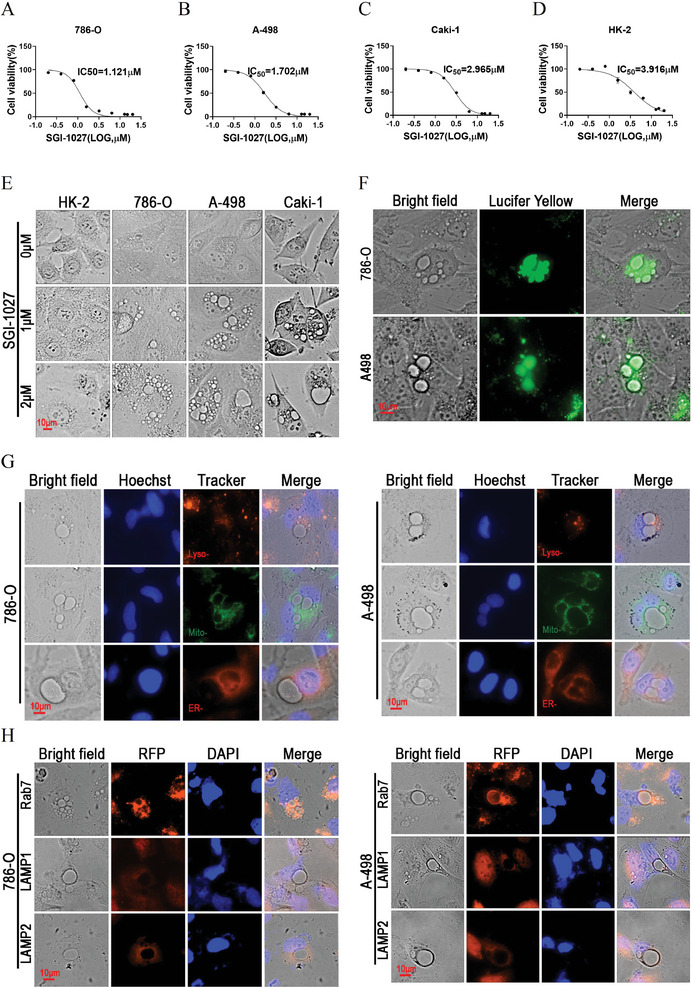
SGI‐1027 induced vacuoles via macropinocytosis in renal cancer cells. A–D) The half maximal inhibitory concentration (IC_50_) of SGI‐1027 in 786‐O, A‐498, Caki‐1 and HK‐2 cells calculated by nonlinear regression. 786‐O, A‐498, Caki‐1 and HK‐2 cells were treated with SGI‐1027 at indicated concentrations for 48 h. E) HK‐2, 786‐O, A‐498, and Caki‐1 cells were treated with 1 × 10^−6^ m or 2 × 10^−6^ m SGI‐1027 for 24 h and living cell images were captured under microscope. F) Lucifer yellow staining of 786‐O and A‐498 cells. 786‐O and A‐498 cells were treated with 1.5 × 10^−6^ m SGI‐1027 and 2 mg mL^−1^ Lucifer yellow for 24 h. G) 786‐O and A‐498 cells were treated with 1.5 × 10^−6^ m SGI‐1027 for 24 h and then incubated with Lyso‐Tracker, Mito‐Tracker and ER‐Tracker for 30 min. Hoechst was used for specifically staining of the nucleus. H) Immunofluorescent staining of Rab7, LAMP1 and LAMP2 in 786‐O and A‐498 cells treated with 1.5 × 10^−6^ m SGI‐1027 for 24 h.

Initially, we assessed the cytotoxicity of SGI‐1027 on renal cancer cells (786‐O, A‐498, Caki‐1) and HK‐2 proximal tubule epithelial cells. SGI‐1027 inhibited the cell viability of all tested cell lines in a dose‐dependent manner (Figure S1, Supporting Information), with respective IC_50_ values of 1.121 × 10^−6^ m, 1.702 × 10^−6^ m, 2.965 × 10^−6^ m and 3.916 × 10^−6^ m in 786‐O, A‐498, Caki‐1 and HK‐2 cells (**Figure** **1**A–D). In addition to its cytotoxic effects, SGI‐1027 demonstrated the ability to induce vacuolization in renal cancer cells. As illustrated in Figure 1E, treatment with SGI‐1027 led to the formation of numerous vacuoles in the cytoplasm of 786‐O, A‐498 and Caki‐1 cells, which tended to fuse and expand. By contrast, HK‐2 cells were less susceptible to the SGI‐1027‐induced vacuolization, exhibiting smaller and fewer vacuoles. To identify the origin of the cytoplasmic vacuoles induced by SGI‐1027, we performed live cell staining using fluorescent tracers to mark lysosomes, mitochondria and endoplasmic reticulum in 786‐O and A‐498 cells. Interestingly, the SGI‐1027‐induced vacuoles did not co‐localize with any of the tested tracers (Figure 1G), indicating that the vacuoles were not derived from lysosomes, mitochondria or endoplasmic reticulum. Since the formation of vacuoles is one of the characteristics of macropinocytosis, a liquid‐phase endocytic process that involves the formation of large vacuoles with some characteristics of late endosomes,^[^ ^24^ ^]^ we investigated whether the SGI‐1027‐induced vacuoles were derived from macropinocytosis by using fluid‐phase tracer Lucifer Yellow and immunofluorescence targeting late endosome marker Rab7. Encouragingly, the fluorescence of Lucifer Yellow and Rab7 were found to accumulate in the vacuoles, suggesting that the vacuoles might originate from macropinocytosis (Figure 1F,H). However, the vacuoles did not colocalize with the lysosome markers LAMP1 and LAMP2 (Figure 1H), indicating that they could not merge with lysosomes.

### 2.2 SGI‐1027‐Induced Cytotoxicity Involved Methuosis in Renal Cancer Cells

**Figure 2:**
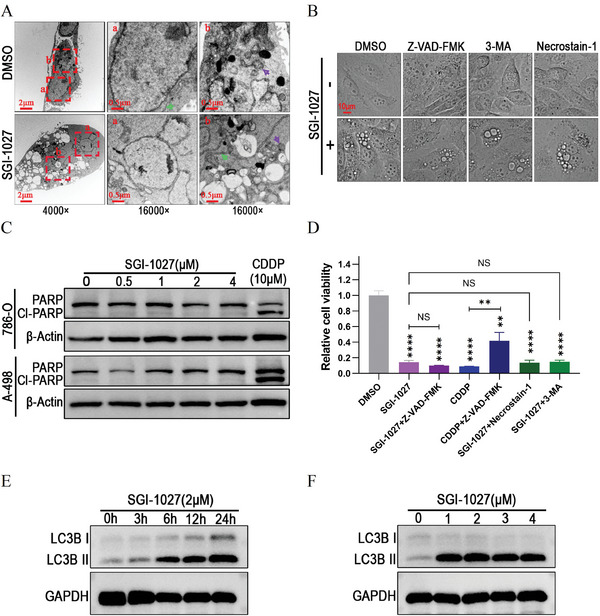
SGI‐1027‐induced cytotoxicity involved methuosis in renal cancer cells. A) 786‐O cells were treated with 1.5 × 10^−6^ m SGI‐1027 for 24 h and then observed under transmission electron microscopy after the preparation of cell sections. The green and purple arrows indicated the endoplasmic reticulum and mitochondria, respectively. B) Living cell images illustrating the vacuolation induced by SGI‐1027 independently on apoptosis, autophagy or necrosis. 786‐O cells were treated with1.5 × 10^−6^ m SGI‐1027, 50 × 10^−6^ m Z‐VAD‐FMK, 1 × 10^−3^ m 3‐MA, 50 × 10^−6^ m necrostatin‐1 or their combinations as indicated for 24 h. C) Typical Western blot showing the expression of PARP and cleaved PARP (Cl‐PARP). 786‐O and A‐498 cells were treated with DMSO (control), SGI‐1027 at indicated concentrations, or 10 × 10^−6^ m cis‐diaminodichloroplatinum (CDDP, positive control) for 48 h. PARP and cleaved PARP (Cl‐PARP) were detected by Western blot, and β‐actin was used as loading control. D) For validating the relationship between SGI‐1027 and apoptosis, 786‐O cells were pretreated with 50 × 10^−6^ m Z‐VAD‐FMK for 2 h and then treated with 2 × 10^−6^ m SGI‐1027 or 10 × 10^−6^ m cis‐diaminodichloroplatinum (CDDP, positive control) for 48 h. For validating the relationship between SGI‐1027 and autophagy or necrosis, 786‐O cells were treated with 2 × 10^−6^ m SGI‐1027, 1 × 10^−3^ m 3‐MA, 50 × 10^−6^ m necrostatin‐1 or their combinations as indicated for 48 h. The cell viability was detected by CCK‐8 assay. NS, not significant; **P < 0.01; ****P < 0.0001. E,F) 786‐O cells were treated with 2 × 10^−6^ m SGI‐1027 for the indicated time or treated with SGI‐1027 for 24 h at the indicated concentrations. LC3B was detected by Western blot, and GAPDH was used as loading control.

Methuosis is a form of cell death characterized by the formation of numerous vacuoles through macropinocytosis and inability of vacuoles to fuse with lysosomes.^[^ ^24^, ^28^ ^]^ To investigate whether methuosis was involved in the cytotoxicity of SGI‐1027, we examined the ultrastructural changes in 786‐O cells treated with SGI‐1027 using transmission electron microscopy. As shown in **Figure** **2**A, SGI‐1027 treatment caused the accumulation of numerous vacuoles bounded by a single membrane in the cells. Meanwhile, the mitochondria and endoplasmic reticulum remained integrated. Notably, the nuclear membrane remained intact and the nuclear chromatin remained diffuse. As methuosis is defined as a type of cell death independent of apoptosis, autophagy and necrosis,^[^ ^24^ ^]^ we next tested whether the vacuolation induced by SGI‐1027 could be prevented by inhibitors of these pathways. The results demonstrated that the formation of vacuolation was not dependent on apoptosis, autophagy, or necrosis, as it could not be prevented by the apoptosis inhibitor ZVAD‐FMK, the autophagy inhibitor 3‐methyladenine (3‐MA) and the necroptosis inhibitor necrostatin‐1 (Figure 2B).

Previous studies have shown that SGI‐1027 can induce apoptosis in hepatoma carcinoma cells.^[^ ^29^ ^]^ To explore the relationship between apoptosis and SGI‐1027 within the vacuolation‐inducing concentration range, we examined the ability of SGI‐1027 to induce cleavage of PARP and its effects on the apoptosis rate in 786‐O and A‐498 cells. Compared to cis‐diaminodichloroplatinum (CDDP), SGI‐1027 hardly induced the cleavage of PARP (Figure 2C). In addition, ZVAD‐FMK could relieve the cytotoxicity of CDDP (*P* < 0.01), but not SGI‐1027 (*P* > 0.05, Figure 2D). The above results proved that SGI‐1027 was a poor apoptosis inducer within the vacuolation‐inducing concentration range. Similarly, necrostatin‐1 could not prevent the cytotoxicity of SGI‐1027 (Figure 2D), which ruled out the possibility of SGI‐1027 being a necroptosis inducer. Subsequently, we explored the relationship between SGI‐1027 and autophagy. We first investigated whether SGI‐1027 could alter the expression of LC3B, an important marker of autophagy.^[^ ^30^ ^]^ As shown in Figure 2E,F, SGI‐1027 caused the accumulation of LC3B‐II by time‐ and dose‐dependent manners, suggesting that SGI‐1027 disrupted autophagic flux of renal cancer cells. However, 3‐MA could not prevent the cytotoxicity of SGI‐1027 (Figure 2D), which excluded autophagy as the cytotoxicity of SGI‐1027. Taken together, these findings suggested that methuosis was involved in the cell death caused by SGI‐1027.

### 2.3 SGI‐1027 Cooperated with Everolimus to Suppress the Proliferation of Renal Cancer Cells

**Figure 3:**
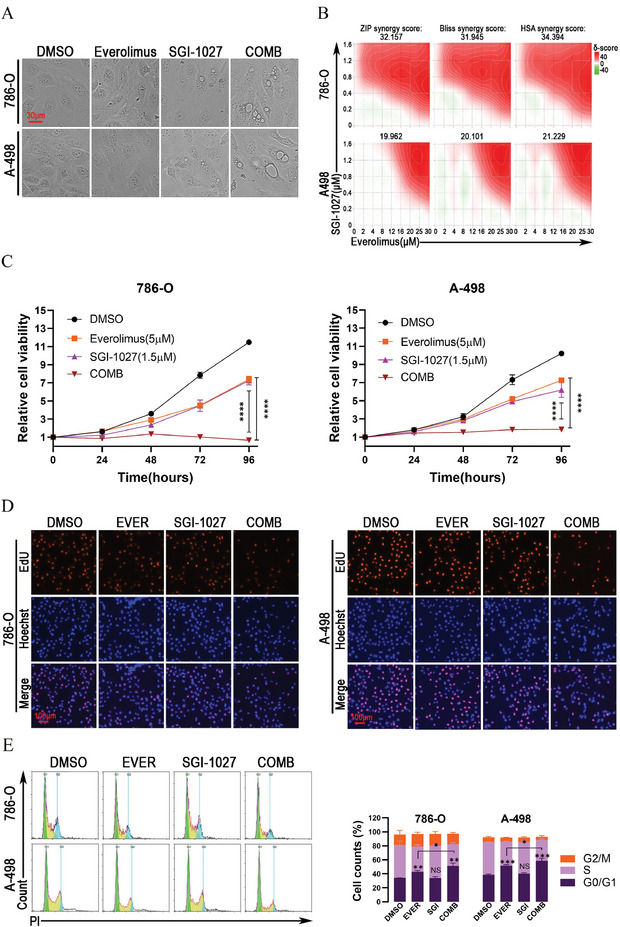
SGI‐1027 and everolimus synergically suppress the proliferation of renal cancer cells. A) Everolimus promoted SGI‐1027‐induced vacuolation in renal cancer cells. 786‐O and A‐498 cells were treated with DMSO (control), 5 × 10^−6^ m everolimus, 1.5 × 10^−6^ m SGI‐1027, and their combination for 12 h. B) Synergy scoring models demonstrating the synergistic effect of SGI‐1027 and everolimus. 786‐O and A‐498 cells were treated with different concentrations of SGI‐1027 and everolimus, or their combination as indicated for 24 h. Subsequently, the synergy scoring models were constructed by Synergyfinder software according to the cell viability. C) CCK‐8 assay for detecting the cell proliferation of 786‐O and A‐498 cells treated with DMSO (control), 5 × 10^−6^ m everolimus, 1.5 × 10^−6^ m SGI‐1027, or their combination for indicated time. D) EdU assay for labeling the cells in the state of DNA synthesis. 786‐O and A‐498 cells were treated with DMSO (control), 5 × 10^−6^ m everolimus, 1.5 × 10^−6^ m SGI‐1027, or their combination for 24 h before EdU staining. E) The synergistic effect of SGI‐1027 and everolimus on the cell cycle of renal cancer cells. 786‐O and A‐498 cells were treated with DMSO (control), 5 × 10^−6^ m everolimus, 1.5 × 10^−6^ m SGI‐1027, or their combination for 24 h before propidium iodide (PI) staining and flow cytometry analysis. NS, not significant; *P < 0.05; **P < 0.01; ***P < 0.001; ****P < 0.0001. EVER, everolimus; SGI, SGI‐1027; COMB, SGI‐1027 combined with everolimus.

SGI‐1027 emerged as a promising candidate in inducing methuosis, but it also captured our attention because of its synergistic effect with everolimus. As shown in **Figure** **3**A, more and larger vacuoles in the SGI‐1027 plus everolimus group were observed compared to the SGI‐1027 group within 12 h. Further analysis demonstrated that their combination exhibited stronger cytotoxicity than the monotherapy group in renal cancer cells (Figure S2A,B, Supporting Information; SGI‐1027 + everolimus vs SGI‐1027). We further constructed the ZIP, Bliss and HAS synergy scoring models to substantiate their synergism (Figure 3B). The results revealed that the interaction between SGI‐1027 and everolimus exhibited synergistic effects, as synergy scores of all models exceeded 10 in both 786‐O and A‐498 cells (Figure 3B). Subsequently, we delved into evaluating their combined cytotoxicity on the proliferation of renal cancer cells. Surprisingly, the combination of SGI‐1027 and everolimus significantly inhibited the proliferation of 786‐O and A‐498 cells within 96 h according to the CCK‐8 assay (*P* < 0.0001, SGI‐1027 + everolimus vs SGI‐1027 or everolimus, Figure 3C). Their combination also significantly reduced the number of EdU‐positive cells compared to monotherapy treatments within 24 h in the EdU assay (*P* < 0.0001, SGI‐1027 + everolimus vs DMSO; *P* < 0.0001 or *P* < 0.001, SGI‐1027 + everolimus vs everolimus; *P* < 0.01, SGI‐1027 + everolimus vs SGI‐1027, Figure 3D). Moreover, 5 × 10^−6^ m of everolimus induced G0/G1 arrest of renal cancer cells (*P* < 0.01, 786‐O or *P* < 0.001, A‐498, Figure 3E), and 1.5 × 10^−6^ m of SGI‐1027 had no significant effect on the cell cycle (*P* > 0.05). However, their combination caused a greater extent of G0/G1 phase arrest compared to everolimus treatment alone (*P* < 0.05, Figure 3E), suggesting that SGI‐1027 enhances the efficacy of everolimus in inducing G0/G1 arrest in renal cancer cells.

### 2.4 SGI‐1027 and Everolimus Synergistically Inhibited the Colony Formation, Migration, and Invasion of Renal Cancer Cells

**Figure 4:**
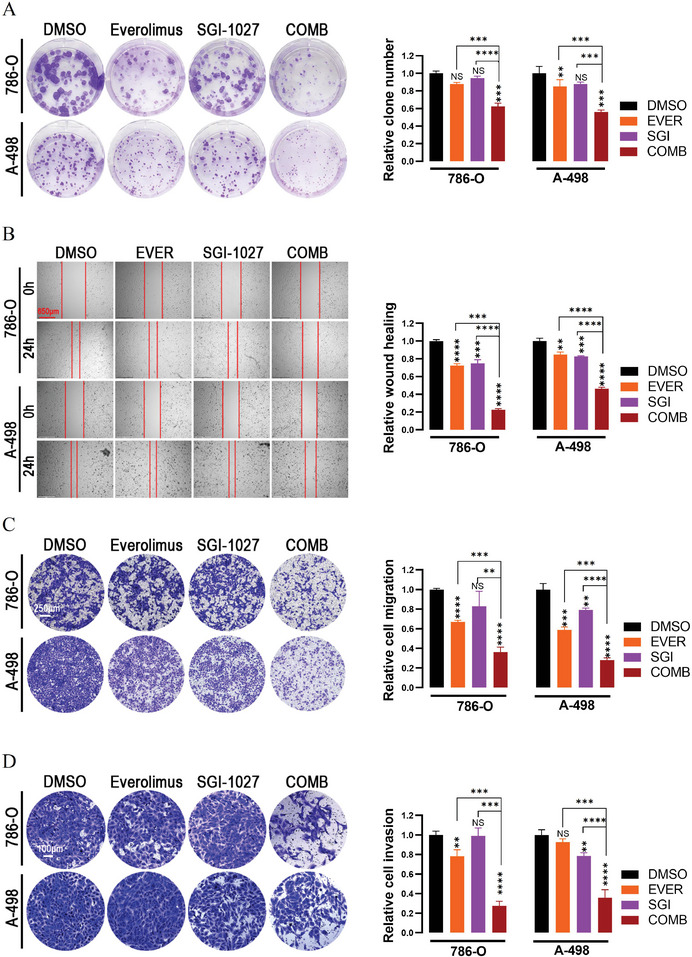
SGI‐1027 and everolimus synergically inhibited the colony formation, migration and invasion in renal cancer cells. A) Colony formation assay detecting the proliferation ability of renal cancer cells. 786‐O and A‐498 cells were stained with crystal violet after incubation with DMSO (control), 1 × 10^−6^ m everolimus, 1 × 10^−6^ m SGI‐1027, or their combination for 14 d. B) Wound‐healing assay and C) transwell migration assay detecting the migration of renal cancer cells. 786‐O and A‐498 cells were treated with 5 × 10^−6^ m everolimus, 1.5 × 10^−6^ m SGI‐1027, or their combination for 24 h in each assay. D) Transwell invasion assay detecting the invasion of renal cancer cells. 786‐O and A‐498 cells were treated with DMSO (control), 5 × 10^−6^ m everolimus, 1.5 × 10^−6^ m SGI‐1027, or their combination for 48 h. NS, not significant; **P < 0.01; ***P < 0.001; ****P < 0.0001. EVER, everolimus; SGI, SGI‐1027; COMB, SGI‐1027 combined with everolimus.

To further explore the synergistical effects of SGI‐1027 and everolimus on the growth of renal cancer cells, we performed colony formation assay. The combination of SGI‐1027 and everolimus showed stronger inhibition on cloning compared to DMSO or their using alone (*P* < 0.001, SGI‐1027 + everolimus vs DMSO; *P* < 0.001, SGI‐1027 + everolimus vs everolimus; *P* < 0.001 or *P* < 0.0001, SGI‐1027 + everolimus vs SGI‐1027, **Figure** **4**A). Considering the key roles of migration and invasion in advanced RCC, we investigated whether the combination of SGI‐1027 and everolimus could synergistically inhibit the migration and invasion abilities of renal cancer cells. In the wound‐healing assay, the combination of everolimus and SGI‐1027 exhibited a greater inhibitory effect on the migration of renal cancer cells compared to their using alone (*P* < 0.001 or *P* < 0.0001, SGI‐1027 + everolimus vs everolimus; *P* < 0.0001, SGI‐1027 + everolimus vs SGI‐1027, Figure 4B). The transwell migration and invasion assays were consistent with the findings from the colony formation and wound healing results (Figure 4C,D). These results confirmed that the combination of SGI‐1027 and everolimus could synergistically inhibit the migration and invasion abilities of renal cancer cells.

### 2.5 SGI‐1027 Cooperated with Everolimus to Induce Apoptosis and GSDME Dependent Pyroptosis

**Figure 5:**
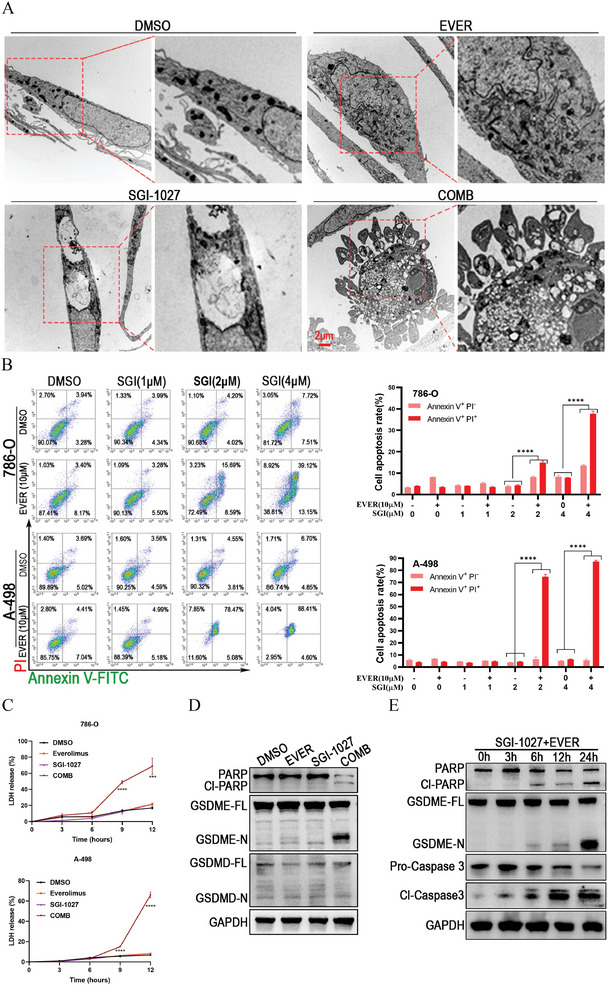
SGI‐1027 cooperated with everolimus to induce apoptosis and GSDME‐dependent pyroptosis. A) The transmission electron microscopy imaging of 786‐O cells treated with DMSO (control), 5 × 10^−6^ m everolimus, 1.5 × 10^−6^ m SGI‐1027, or their combination for 24 h. B) Annexin V and propidium iodide (PI) staining for 786‐O and A‐498 cells treated with DMSO (control), 10 × 10^−6^ m everolimus, different concentrations of SGI‐1027 and their combinations as indicated. C) LDH release assay detecting the LDH releasing of renal cancer cells treated with DMSO (control), 5 × 10^−6^ m everolimus, 1.5 × 10^−6^ m SGI‐1027, or their combination for indicated time. D) A‐498 cells were treated with DMSO (control), 5 × 10^−6^ m everolimus, 2 × 10^−6^ m SGI‐1027, or their combination for 24 h. PARP, cleaved PARP (Cl‐PARP), GSDME, GSDMD were detected by Western blot, and GAPDH was used as loading control. E) A‐498 cells were treated with the combination of 5 × 10^−6^ m everolimus and 2 × 10^−6^ m SGI‐1027 for indicated time. PARP, cleaved PARP, GSDME, pro‐Caspase 3, cleaved Caspase 3 (Cl‐Caspase 3) were detected by Western blot. GAPDH was used as loading control. ***P < 0.001; ****P < 0.0001. EVER, everolimus; SGI, SGI‐1027; COMB, SGI‐1027 combined with everolimus.

To explore the form of cell death caused by the combination of everolimus and SGI‐1027, we investigated the morphological changes of dead cells under transmission electron microscopy. As shown in **Figure** **5**A, after treatment with SGI‐1027 in combination with everolimus for 24 h, the dead cells were characterized by cellular swelling and rounding, large bubbles emanating from the plasma membrane, chromatin condensation, as well as hypervacuolated cytoplasm. Subsequently, we confirmed the nature of cell death by flow cytometry analysis of propidium iodide (PI) and annexin V staining. As shown in Figure 5B, the combination of SGI‐1027 and everolimus significantly induced cell death, with most dead cells proceeding directly to the annexin V and PI double‐positive stage within 24 h (*P* < 0.0001, 2 × 10^−6^ or 4 × 10^−6^ m SGI‐1027 + everolimus vs SGI‐1027). We further performed the lactate dehydrogenase (LDH) releasing assay to explore whether cell membrane integrity was lost during cell death. As shown in Figure 5C, SGI‐1027 or everolimus alone did not increase LDH release, but their combination significantly increased LDH release after 9 h (*P* < 0.0001, SGI‐1027 + everolimus vs DMSO), reaching over 60% after 12 h (*P* < 0.001 in 786‐O cells, *P* < 0.0001 in A‐498 cells, SGI‐1027 + everolimus vs everolimus or SGI‐1027), suggesting that cell membrane damage occurred at an early stage of cell death. Given that the early loss of plasma membrane integrity is a hallmark of pyroptosis, we subsequently investigated the relationship between the combination of SGI‐1027 and everolimus and pyroptosis. Interestingly, the combination of SGI‐1027 and everolimus induced cleavage of PARP and GSDME, rather than GSDMD (Figure 5D), indicating that their combination induced apoptosis and GSDME‐dependent pyroptosis in renal cancer cells. Additionally, the cleavages of PARP, GSDME, and Caspase 3 were observed after 6 h of treatment (Figure 5E), which was consistent with the results of the LDH release assay. Taken together, these findings suggest that SGI‐1027 cooperates with everolimus to induce apoptosis and pyroptosis in renal cancer cells.

### 2.6 RNA Sequencing Analysis Unveiled the Association between the Synergistic Effect and Lysosome

**Figure 6:**
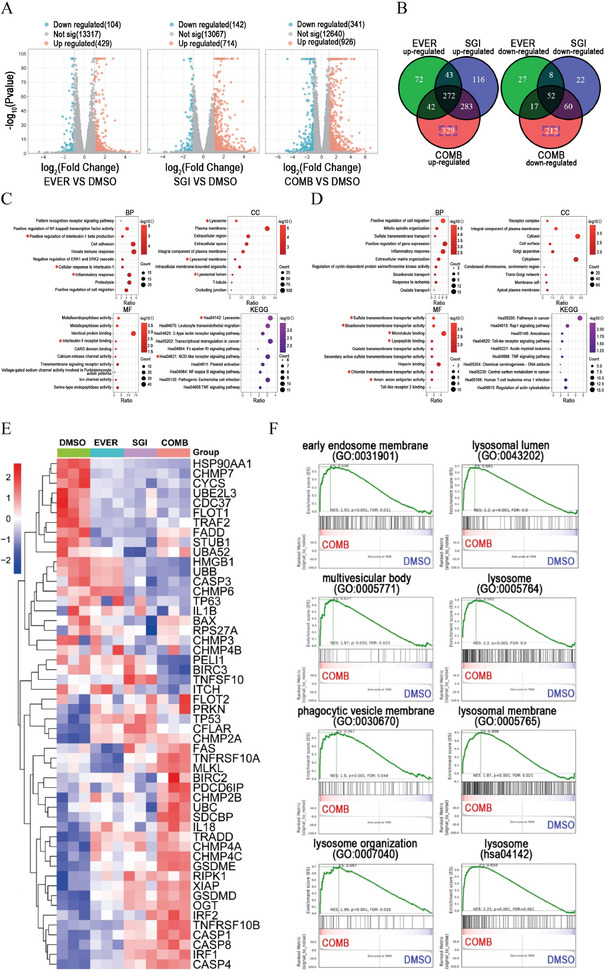
RNA sequencing analysis unveiled the association between the synergistic effect and lysosome. A) The volcano plots for everolimus group, SGI‐1027 group, and SGI‐1027 combined with everolimus group. B) Venn plots of upregulated or downregulated DEGs in everolimus group, SGI‐1027 group and SGI‐1027 combined with everolimus group. C,D) GO and KEGG enrichment analyses for up‐regulated DEGs C) and down‐regulated DEGs D) unique to the combination group. BP, biological process; CC, cellular component; MF, molecular function; KEGG, Kyoto Encyclopedia of Genes and Genomes. E) Heatmap of the expression of pyroptosis‐related genes in the control group (DMSO), everolimus, SGI‐1027, and combination treatment group. F) GSEA enrichment analyses for gene sets related to cytophagy and lysosome in combination group. ES, enrichment score; NES, normalized enrichment score. EVER, everolimus; SGI, SGI‐1027; COMB, SGI‐1027 combined with everolimus.

Next, we conducted RNA sequencing to explore the mechanism underlying the combination of SGI‐1027 and everolimus in inducing pyroptosis. We identified 429 upregulated and 104 downregulated DEGs in the everolimus group, 714 upregulated and 142 downregulated DEGs in the SGI‐1027 group, and 926 upregulated and 341 downregulated DEGs in the combination group, respectively (**Figure** **6**A, Table S1, Supporting Information and GSE242110 online). Gene enrichment analysis for the DEGs between the SGI‐1027 group and control group indicated an association with “phagosome” consistent with the cytotoxicity of SGI‐1027 in inducing methuosis (Figure S3B, Supporting Information). Additionally, the DEGs between the combination group and control group were associated with the “Nod‐like receptor signaling pathway” and “lysosome” (Figure S3C, Supporting Information). However, these terms were not enriched in the everolimus group. (Figure S3A, Supporting Information). To accurately investigate the synergism of SGI‐1027 and everolimus, we identified 329 upregulated and 212 down‐regulated DEGs unique to the combination group using the Venn tool (Figure 6B). Gene enrichment analysis for the up‐regulated DEGs showed associations with multiple pyroptosis‐related signaling pathways, including “positive regulation of interleukin‐1 beta production”, “cellular response to interleukin‐1,” “inflammatory response,” “interleukin‐1 receptor binding” and “NOD‐like receptor signaling pathway” (Figure 6C). The heatmap of pyroptosis and inflammatory response‐related genes also suggested the activation of pyroptotic signaling in the combination group (Figure 6E and Figure S4, Supporting Information). Most importantly, lysosome‐related signaling pathways ranked first in both cellular component (CC) and KEGG enrichment analyses, underscoring the significant relationship between the combination of SGI‐1027 and everolimus with lysosome and pyroptosis (Figure 6C). Additionally, gene enrichment analysis for the 212 downregulated DGEs showed that they were related to “sulfate transmembrane transporter activity,” “bicarbonate transmembrane transporter activity,” “microtubule binding,” “lipopeptide binding,” “chloride transmembrane transporter activity,” and “anion antiporter activity” (Figure 6D), which play regulatory roles in the process of pinocytosis and micropinocytosis. Furthermore, the GSEA enrichment analysis indicated that “early endosome membrane,” “multivesicular body,” “phagocytic vesicle membrane” and lysosome‐related signaling pathways, including “lysosome organization,” “lysosome,” “lysosomal membrane” and “lysosomal lumen” were activated in the combination group (Figure 6F). In summary, these findings strongly support the ability of SGI‐1027 in inducing cell methuosis and indicated the potential role of lysosome in the synergistic action of SGI‐1027 and everolimus.

### 2.7 SGI‐1027 Cooperated with Everolimus to Induce Lysosomal Membrane Permeability in Renal Cancer Cells

**Figure 7:**
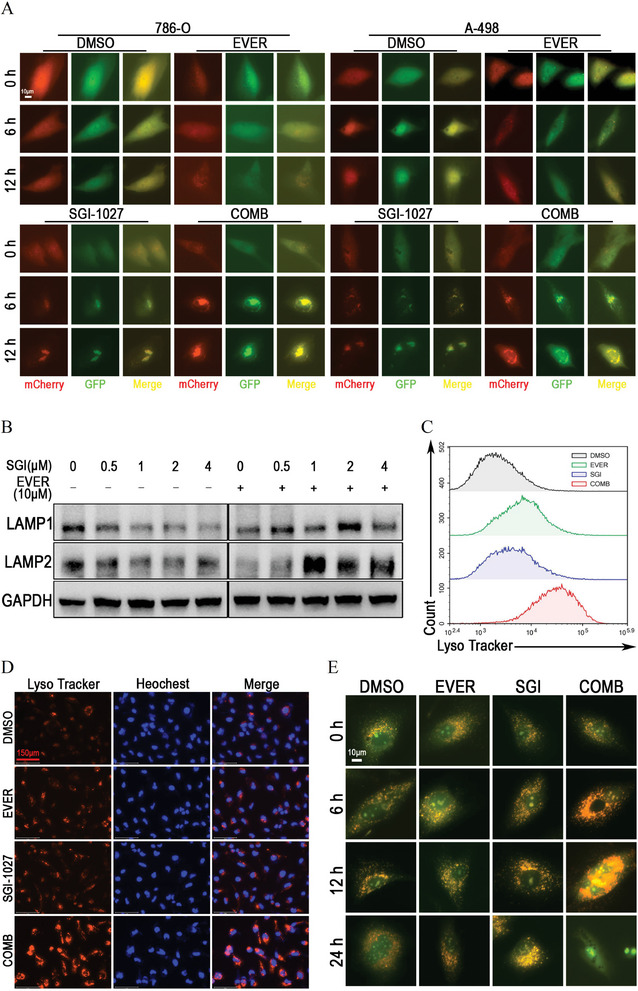
SGI‐1027 cooperated with everolimus to induce lysosomal membrane permeability (LMP) in renal cancer cells. A) Detection of autophagy flux in renal cancer cells. 786‐O and A‐498 cells were infected by Ad‐mCherry‐GFP‐LC3B, after which cells were treated with DMSO (control), 5 × 10^−6^ m everolimus, 2 × 10^−6^ m SGI‐1027, or their combination for indicated time. B) A‐498 cells were treated with 10 µM everolimus, different concentrations of SGI‐1027, or their combination as indicated for 24 h. LAMP1 and LAMP2 were detected by Western blot. GAPDH was used as loading control. C) Flow cytometry analysis and D) Lyso‐Traker staining for A‐498 cells treated with DMSO (control), 5 × 10^−6^ m everolimus, 2 × 10^−6^ m SGI‐1027, or their combination for 12 h. E) Acridine orange staining for A‐498 cells treated with DMSO (control), 5 × 10^−6^ m everolimus, 2 × 10^−6^ m SGI‐1027, or their combination for indicated time. EVER, everolimus; SGI, SGI‐1027; COMB, SGI‐1027 combined with everolimus.

Next, we observed whether the synergistic effect of SGI‐1027 with everolimus is related to the inhibition of DNMT1. Interestingly, another DNMT1 inhibitor, 5‐aza‐2′‐deoxycytidine, failed to induce vacuolization in renal cancer cells and did not exhibit significant synergistic effects with everolimus (Figure S5, Supporting Information). Based on the insights from RNA sequencing analysis, we subsequently investigated the role of lysosome in the cell death caused by the combination of SGI‐1027 and everolimus. First, we assessed the effect of SGI‐1027 in combination with everolimus on the formation of autolysosomes by using an adenovirus expressing mCherry‐GFP‐LC3B fusion protein.^[^ ^23^ ^]^ As shown in **Figure** **7**A, SGI‐1027 induced accumulation of yellow signals in renal cancer cells, and everolimus amplified this effect within a short time, suggesting that the formation of autolysosomes was disturbed. Moreover, SGI‐1027 induced the degradation of LAMP1 and LAMP2, but it inversely caused accumulation of LAMP1 and LAMP2 in the presence of everolimus (Figure 7B). These results suggested that their combination disrupted lysosomal turnover and caused enlargement of lysosomes.^[^ ^31^, ^32^ ^]^ We next detected the lysosome by flow cytometry and Lyso‐Tracker staining, finding that SGI‐1027 combined with everolimus enhanced the fluorescence signals of lysosomes within 12 h (Figure 7C,D). We further clarified the lysosomal changes using acridine orange (AO) staining. Interestingly, the results showed that SGI‐1027 combined with everolimus caused the expanding of cytoplastic acidic region within 12 h, but the acidic region disappeared after 24 h (Figure 7E), which suggested the triggering of LMP.^[^ ^33^ ^]^

### 2.8 Targeting LMP and GSDME‐Dependent Pyroptosis Manifested Feasibility for RCC Therapy

**Figure 8:**
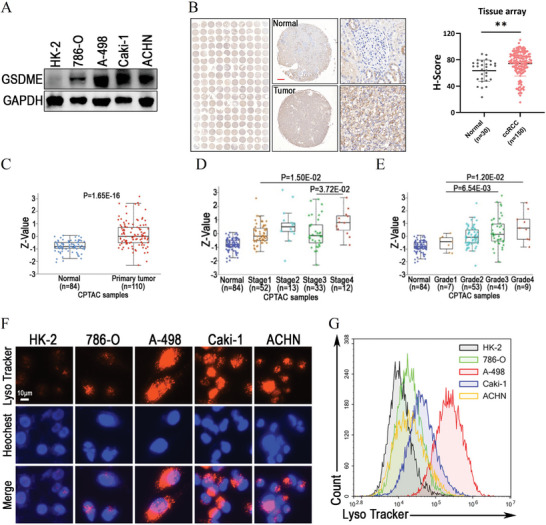
Targeting LMP and GSDME‐dependent pyroptosis manifests feasibility for RCC therapy. A) Detection of the endogenous expression of GSDME in HK‐2 and renal cancer cells as indicated by Western blot. GAPDH was used as loading control. B) GSDME protein expression was examined utilizing a ccRCC tissue array consisting of 30 normal kidney tissues and 150 tumor tissues. GSDME protein level was significantly higher in ccRCC tissues compared to normal kidney tissues. **P < 0.01. C) The differential expression of GSDME protein between clear cell renal cell carcinoma (ccRCC) and normal kidney tissues in CPTAC database. D,E) The expression of GSDME protein in different stage and grade of ccRCC in CPTAC database. F,G) Lyso‐Tracker staining and flow cytometry analysis for HK‐2, 786‐O, A‐498, Caki‐1, and ACHN cells.

We further analyzed the differences in cytotoxicity between the combination of SGI‐1027 and everolimus against HK‐2 and renal cancer cells. The results demonstrated that 4 × 10^−6^ m SGI‐1027 combined with 10 × 10^−6^ m everolimus resulted in more than 60% cell death in Caki‐1 and ACHN cells, but less than a 20% increase in cell death in HK‐2 cells. (Figure S6A,B, Supporting Information). Under the same concentration combination of SGI‐1027 and everolimus, the morphological changes indicative of pyroptosis were observed in 786‐O, A‐498, Caki‐1, and ACHN cells, but not in HK‐2 cells (Figure S6C, Supporting Information). Furthermore, with everolimus fixed at 5 × 10^−6^ m, the MOS (margin of safety) for the combination of SGI‐1027 and everolimus were 2.04 and 1.65 in 786‐O and A‐498 cells, respectively (Figure S7A–C, Supporting Information). These results indicated that HK‐2 cells might resist LMP and GSDME‐dependent pyroptosis induced by the combination treatment. We therefore examined the endogenous expression of GSDME in HK‐2 cells and renal cancer cells. The result demonstrated that 786‐O, A‐498, Caki‐1 and ACHN cells expressed GSDME at extremely higher levels than HK‐2 cells (**Figure** **8**A). By analyzing the protein expression levels of GSDME in tissue microarrays comprising 150 ccRCC cases and 30 normal kidney cases, we demonstrated a significant increase in GSDME expression in ccRCC (Figure 8B, ccRCC vs normal kidney, *P* < 0.01). We also analyzed proteomic data from the CPTAC database to explore the expression of GSDME in clear cell renal cell carcinoma (ccRCC) tissues. The results indicated that GSDME was highly expressed in ccRCC tissues compared to normal kidney tissues (*P* = 1.65E‐16, Figure 8C). Furthermore, GSDME expression was correlated with tumor stage (*P* = 1.50E‐02, Stage4 vs Stage1; *P* = 3.72E‐02, Stage4 vs Stage3, Figure 8D) and grade (*P* = 6.54E‐03, Grade3 vs Grade1; *P* = 1.20E‐02, Grade4 vs Grade1, Figure 8E) of ccRCC, suggesting that GSDME could be a biomarker for advanced renal carcinoma. Existing research suggests that high expression level of endogenous GSDME makes cells more prone to undergoing GSDME‐dependent pyroptosis.^[^ ^25^ ^]^ This may explain the heterogeneity in the toxicity of SGI‐1027 combined with everolimus between renal cancer cells and HK‐2 cells. For further validation, we knocked down GSDME expression in 786‐O and A‐498 cells and found that it increased the resistance of renal cancer cells to the combined toxicity of SGI‐1027 and everolimus (Figure S7D–F, Supporting Information). Additionally, since the quantity and activity of lysosomes in tumor cells can affect their susceptibility to LMP,^[^ ^34^ ^]^ we further investigated the differences in lysosomal activity between renal cancer cells and HK‐2 cells using Lyso‐Tracker staining. Remarkably, the lysosomal signals of 786‐O, A‐498, Caki‐1 and ACHN renal cancer cells were significantly higher than that of HK‐2 cells (Figure 8F), which was also confirmed by the results of flow cytometry (Figure 8G). Therefore, our findings indicate that renal cancer cells exhibit high expression level of GSDME and increased lysosomal activity. Based on these, the combination of SGI‐1027 and everolimus could be a promising treatment option for advanced RCC due to its beneficial effects in inducing LMP and GSDME‐dependent pyroptosis.

### 2.9 SGI‐1027 Combined with Everolimus Exerted Synergetic Antitumor Effects In Vivo

**Figure 9:**
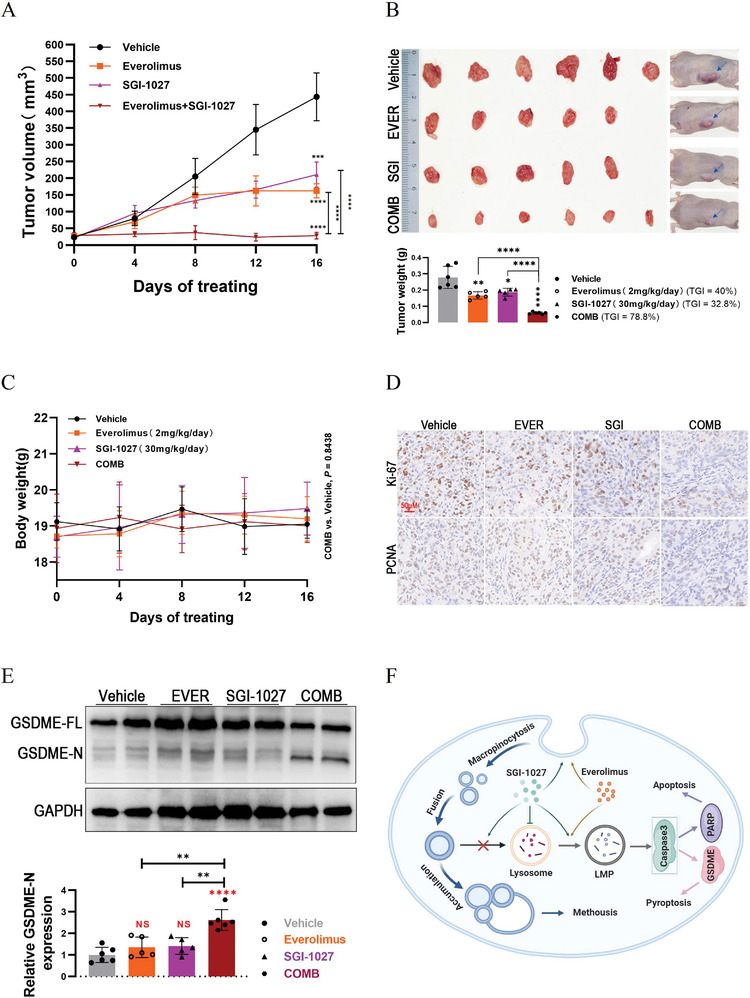
SGI‐1027 combined with everolimus exerted synergetic anti‐tumor effects in vivo. A) Tumor volume of subcutaneous xenograft models during treatment. Carboxymethyl cellulose (vehicle), SGI‐1027 (SGI, 30 mg kg^−1^ per day), everolimus (EVER, 2 mg kg^−1^ per day) and their combination (COMB) were administered once daily by oral gavage. B) Image and tumor weight of the subcutaneous xenograft tumors harvested from nude mice models. TGI, tumor growth inhibition value. C) Body weight of subcutaneous xenograft models during 16 d of treatment. COMB, SGI‐1027 combined with everolimus. D) IHC staining analysis of Ki‐67 and PCNA for the harvested subcutaneous tumors. EVER, everolimus; SGI, SGI‐1027; COMB, SGI‐1027 combined with everolimus. E) Western blot analysis to detect the cleavage state of GSDME in subcutaneous tumors. NS, not significant; *P < 0.05; **P < 0.01; ***P < 0.001; ****P < 0.0001. F) A possible schematic model for cell death induced by SGI‐1027 in combination of everolimus suggested by the present study: SGI‐1027 induces vacuolation by macropinocytosis and inhibits the fusion of vacuoles and lysosomes. Everolimus enhances the process of macropinocytosis and cooperates with SGI‐1027 to induce LMP, subsequently leading to apoptosis and GSDME dependent pyroptosis.

We finally established a nude mice xenograft model using A‐498 cells to evaluate the in vivo anti‐tumor activity of SGI‐1027, everolimus, and their combination. As shown in **Figure** **9**A,B, treatment with 30 mg kg^−1^ per day of SGI‐1027 or 2 mg kg^−1^ per day of everolimus for 16 d significantly inhibited tumor growth compared to the vehicle group (tumor volume: *P* < 0.001 and *P* < 0.0001, SGI‐1027 or everolimus vs vehicle; tumor weight: *P* < 0.05 and *P* < 0.01, SGI‐1027 or everolimus vs vehicle). Surprisingly, the combination of SGI‐1027 and everolimus showed even stronger suppression of tumor growth compared to either agent alone (tumor volume and weight: *P* < 0.0001, SGI‐1027 + everolimus vs SGI‐1027/everolimus/vehicle). Based on the tumor weight after 16 d of treatment, the tumor growth inhibition value (TGI) of everolimus group and SGI‐1027 group was 40% and 32.8%, respectively (Figure 9B). By contrast, the TGI of the combination group was 78.8%, which suggested that everolimus combined with SGI‐1027 had a better synergistic anti‐tumor effect in vivo. Importantly, the combination treatment did not result in significant weight loss after 16 d of treatment (*P* > 0.05, SGI‐1027 + everolimus vs SGI‐1027/everolimus/Vehicle, Figure 9C). Moreover, the expression levels of Ki‐67 and PCNA in subcutaneous tumors of combination group were lower than those of everolimus group and SGI‐1027 group (Figure 9D). Most importantly, we observed a significant increase in GSDME cleavage in subcutaneous tumors in the combination therapy group (Figure 9E and *P* < 0.0001, SGI‐1027 + everolimus vs vehicle; *P* < 0.01, SGI‐1027 + everolimus vs everolimus or SGI‐1027), suggesting that SGI‐1027 combined with everolimus can promote tumor regression in vivo by inducing pyroptosis. Overall, our results suggest that SGI‐1027 could inhibit tumor growth in vivo, and its combination with everolimus exerts a potent tumor‐suppressive effect by inducing pyroptosis with ideal tolerability.

## 3 Discussion

Rapid progress has been made in the treatment of metastatic RCC in clinical. These treatments are primarily categorized based on their mechanisms of action into tyrosine kinase inhibitors (TKI) (e.g., sorafenib, sunitinib), mTOR inhibitors (e.g., everolimus), and immune checkpoint inhibitors (e.g., nivolumab). For patients who experienced treatment failure with sorafenib or sunitinib, the median PFS with second‐line everolimus treatment was 5.4 months, with a 69% reduction in the risk of disease progression.^[^ ^11^ ^]^ Additionally, a multicenter clinical study confirmed the efficacy and safety of everolimus as a second‐line targeted therapy after TKI treatment failure, with a disease control rate of 61%, a median PFS of 6.9 months, and a clinical benefit rate of 66%.^[^ ^35^ ^]^ However, the issue of everolimus resistance has yet to be resolved. Given these, the identification of new drugs and combination regimens targeting multiple factors is necessary. In this study, we identified SGI‐1027 as a methuosis inducer in renal cancer cells. When combined with everolimus, SGI‐1027 exerted strong lethality by inducing LMP and GSDME‐dependent pyroptosis. A possible schematic model for cell death induced by the combination of SGI‐1027 and everolimus is shown in Figure 9F. Briefly, SGI‐1027 induces vacuolation by macropinocytosis and inhibits the fusion of vacuoles and lysosomes. Everolimus enhances the process of macropinocytosis and cooperates with SGI‐1027 to induce LMP, which subsequently leading to apoptosis and GSDME‐dependent pyroptosis. These findings suggest potential new strategies for everolimus resistance and the management of advanced RCC.

Most existing antitumor drugs induce apoptosis as the main working positively mechanism. However, evasion of apoptosis has been identified as a hallmark of cancer,^[^ ^36^ ^]^ making tumors less susceptible to apoptotic signals and eventually leading to drug resistance.^[^ ^37^ ^]^ Recently, methuosis has been proposed as a nonapoptotic cell death mechanism that could guide tumor therapy for overcoming anti‐apoptotic drug resistance.^[^ ^38^ ^]^ Therefore, identifying new methuosis inducers and evaluating their anti‐tumor activity are necessary for the development of novel drugs. While many methuosis inducers have been identified in vitro studies in recent years, few studies have evaluated their efficacy and side effects in vivo. In our study, we demonstrated that SGI‐1027 showed higher cytotoxicity in renal cancer cells but less in HK‐2 cells. We also verified its antitumor activity in vivo and found it to be safe and well‐tolerated. Therefore, we believe that SGI‐1027 holds promise in the design and development of novel anti‐tumor drugs for overcoming anti‐apoptotic drug resistance. Additionally, the inability of vacuoles to fuse with lysosome is a hallmark of methuosis, and a previous study has proposed that LMP may be associated with the occurrence of methuosis.^[^ ^27^ ^]^ In our study, everolimus enhanced SGI‐1027‐induced vacuolation, which occurred simultaneously with GSDME cleavage. These results suggest that methuosis is involved in the combined toxicity, and LMP may be a key factor mediating the transition from methuosis to pyroptosis.

Although GSDME expression is generally silenced in most cancer cells,^[^ ^39^ ^]^ a recent study has reported that GSDME is a potential clinical target and prognostic biomarker for ccRCC.^[^ ^40^ ^]^ In our study, we found that GSDME was highly expressed in renal cancer cells compared to human proximal tubule epithelial cells. Additionally, GSDME was highly expressed in ccRCC tissues and positively correlated with the stage and grade of ccRCC according to the CPTAC proteomics database. These results support the idea of targeting GSDME‐dependent pyroptosis as a potential therapy for advanced RCC. Additionally, consistent with previous research findings, we found that the lysosomal activity in renal cancer cells was significantly increased, making them more susceptible to LMP.^[^ ^34^ ^]^ Previous studies have shown that LMP can induce GSDME‐dependent pyroptosis in human retinal pigment epithelial cells (ARPE‐19 cells),^[^ ^33^ ^]^ but no study investigating the relationship between LMP and GSDME‐dependent pyroptosis in tumor cells. In this study, we confirmed through in vitro and in vivo experiments that SGI‐1027 combined with everolimus induces LMP and GSDME‐dependent pyroptosis in renal cancer cells. The upregulation of GSDME expression and the increase in lysosomal activity in renal cancer cells will create a greater therapeutic window for the combined treatment.

In conclusion, this study first identified SGI‐1027 as a methuosis inducer in RCC, and SGI‐1027 combined with everolimus could exert powerful lethal effect on RCC cells by inducing LMP, providing new solution to the treatment of advanced RCC, everolimus resistance, as well as GSDME‐positive malignant tumors.

## 4 Experimental Section

### Cell Culture

Human renal cancer cells (786‐O, A‐498, Caki‐1, and ACHN) and human proximal tubule epithelial cells HK‐2 were obtained from Procell (Wuhan, China). 786‐O cells were cultured in Roswell Park Memorial Institute (RPMI) 1640 medium (Cat#C11875500CP, Gibco, USA). A‐498, ACHN, and HK‐2 cells were cultured in Dulbecco's Modified Eagle Medium (DMEM, Cat#L110KJ, BasalMedia, Shanghai, China). Caki‐1 cells were cultured in McCoy's 5A medium (Cat#PM150710, Procell, Wuhan, China). All culture media contained 10% (v/v) fetal bovine serum, 100 U mL^−1^ penicillin, and 100 µg mL^−1^ streptomycin. The cells were maintained at 37 °C in 5% CO_2_

### Chemicals and Reagents

SGI‐1027 (Cat#HY‐13962), everolimus (Cat#HY‐10218), cis‐diaminodichloroplatinum (CDDP, Cat#HY‐17394), Z‐VAD‐FMK (Cat#HY‐16658B), 3‐methyladenine (3‐MA, Cat#HY‐19312), 5‐aza‐2′‐deoxycytidine (Cat#HY‐A0004) and necrostatin‐1(Cat#HY‐15760) were purchased from MedChemExpress (MCE, USA). Dimethyl sulfoxide (DMSO, Cat# 60313ES60) was purchased from Yeasen Biotechnology (Shanghai, China). Phenylmethylsulfonyl fluoride (Cat#ST505), RIPA lysis buffer (Cat#P0013B), proteinase inhibitor (Cat#P1005), and phosphatase inhibitor (Cat#P1045) were obtained from Beyotime Biotechnology (Shanghai, China)

### Cell Viability

CCK‐8 cell proliferation assay kit was purchased from Uelandy Biotechnology (Cat#C6005, Suzhou, China). Cells were seeded in 96‐well plates and allowed to adhere and recover morphology before replacing the medium with drug‐containing culture medium. After incubation for appropriate time, cell viability was determined using the CCK‐8 assay kit according to the manufacturer's instructions. The absorbance at 450 nm was detected by a microplate reader (Synergy H1, BioTek, USA)

### Live Cell Imaging

ER‐Tracker red (Cat#C1041S), Lyso‐Tracker red (Cat#C1046), and Mito‐Tracker green (Cat#C1048) were obtained from Beyotime Biotechnology. Lucifer yellow CH dilithium salt (Cat#L0259‐25MG) was purchased from Sigma‐Aldrich (St. Louis, MO, USA). Acridine orange (Cat#HY‐101879) was purchased from MedChemExpress. For live cell staining, cells were seeded at 5 × 10^4^ per well in a 24‐well plate and treated with SGI‐1027 for 24 h. Subsequently the cells were incubated with ER‐Tracker red, Lyso‐Tracker red, Mito‐Tracker green, Lucifer yellow or acridine orange according to the manufacturer's instructions. All images were captured using a fluorescence microscope (Thermo Fisher, EVOS M7000, USA)

### Immunofluorescence Staining

Cells were fixed with 4% (v/v) paraformaldehyde for 15 min and permeabilized with 0.1% Triton X‐100 for 20 min, followed by blocking with 5% BSA for 1 h. Then, the cells were incubated with Rab7 (Cat#9367, Cell Signaling Technology), LAMP1 (Cat#A16894, Abclonal Technology), and LAMP2 (Cat#A22482, Abclonal Technology) antibodies overnight at 4 °C. After washing for three times with PBS, the cells were incubated with Alexa Fluor 555‐conjugated anti‐rabbit IgG (Cat#ab150078, Abcam) for 1 h. Subsequently, the cells were stained with DAPI staining solution (Cat#C1005, Beyotime Biotechnology). All images were captured under fluorescence microscope (Thermo Fisher, EVOS M7000, USA).

### Transmission Electron Microscopy

Cells were fixed with 2.5% (v/v) glutaraldehyde at room temperature for 30 min and then overnight at 4 °C. After washing for three times with 0.1 m PBS, the cells were fixed with 1% OsO4 in 0.1 m PBS (pH 7.4) at 4 °C for 2 h. Subsequently, the cells were dehydrated with a gradient of ethanol (30%, 50%, 70%, 90%, 100%, and 100%) and embedded in Spurr's resin. Ultrathin sections were cut and placed on copper grids, after which the sections were stained with uranyl acetate or lead citrate and observed under transmission electron microscopy (Hitachi H‐7650, Japan).

### Drug Interaction Evaluation

Zero interaction potency (ZIP) model, Bliss independence model, and highest single agent (HSA) model were used to analyze drug combination data between SGI‐1027 and everolimus.^[^ ^41^ ^]^ The ZIP model was applied to compare the change in potency (impact at a specific dose level) of the dose–response curves between individual medications and their combinations to capture the drug interaction interactions. The Bliss independence model, which assumes a stochastic process in which two drugs act independently, can be used to calculate the expected joint effect based on the probability of independent events. The HSA model considers the expected combined effect to be the maximum of a single drug response at the corresponding concentration. SGI‐1027 and everolimus were combined at non‐fixed doses. The inhibition rate of each concentration combination was analyzed by CCK‐8 assay, and the synergy scores of each reference model were calculated by SynergyFinder software (version 2.0).^[^ ^42^ ^]^ The calculated synergy scores under −10, from −10 to 10 and above 10 indicate antagonism, additivity, and synergism, respectively.

### Colony Formation Assay

Cells were seeded in six‐well plates (500 cells per well) and incubated with drug‐containing culture medium for 2 to 3 weeks, after which cells were fixed with 4% (v/v) paraformaldehyde and stained with 0.1% crystal violet.

### EdU Assay

The EdU cell proliferation kit with Alexa Fluor 555 (Cat#C0075) and Hoechst 33258 (Cat#C1011) for nuclear staining was purchased from Beyotime Biotechnology. About 1 × 10^4^ cells were seeded in 96‐well plates and incubated with drug‐containing culture medium for 24 h, after which cells were fixed with 4% (v/v) paraformaldehyde and stained with EdU and Hoechst according to the manufacturer's instructions. The images were captured under a fluorescence microscope (Thermo Fisher, EVOS M7000).

### Cell Migration and Invasion

In wound‐healing assay, cells were seeded in a six‐well plate and incubated overnight. A 200 µL pipette tip was used to create a scratch in the middle of each well, followed by replacing the serum‐free media with or without drugs. After 24 h, images of the scratched area were collected. Transwell migration plate (Cat#3422, Corning, NY, USA) and transwell invasion plate (Cat#354480, Corning) were used to performed migration and invasion assay according to the manufacturer's instructions. All images were collected under a fluorescence microscope (Thermo Fisher, EVOS M7000, USA).

### Immunoblotting

After drug treatment, cells were lysed in RIPA buffer containing PMSF, protease inhibitor, and phosphatase inhibitor. For subcutaneous tumors, the minced tissues were mixed with RIPA buffer and homogenized. The supernatant was obtained after thorough lysis and centrifugation, and the protein concentration was measured using bicinchoninic acid assay (BCA, Cat#23227, ThermoFisher Scientific, USA). Equal amounts of protein were loaded onto a sodium dodecyl sulfate‐polyacrylamide gel electrophoresis (SDS‐PAGE) gel for electrophoresis, followed by transfer of the proteins onto a PVDF membrane (Cat#03010040001, Roche, Basel, Switzerland). After incubation with specific primary and secondary antibodies, protein was detected by ChemiScope 6200 (Clinx, Shanghai, China). Antibodies against GAPDH (Cat#2118), LC3B (Cat#3868), β‐Actin (Cat#3700), PARP (Cat#9542), Caspase‐3 (Cat#9662) and cleaved Caspase‐3 (Cat#9661) were purchased from Cell Signaling Technology (Beverly, USA). Antibodies against DNMT1 (Cat#A22455), DNMT3A (Cat#A2065), DNMT3B (Cat#A22658), LAMP1 (Cat#A16894) and LAMP2 (Cat#A22482) were obtained from Abclonal Technology (Wuhan, China). Antibodies against GSDMD (Cat#ab219800) and GSDME (Cat#ab19859) were purchased from Abcam (Cambridge, UK).

### RNA Sequencing

786‐O cells were incubated with 1.5 × 10^−6^ m SGI‐1027, 5 × 10^−6^ m everolimus or their combination, or an equal volume of dimethylsulfoxide (DMSO) as a control group. After 24 h of treatment, total RNA was extracted using the TRIzol reagent according to the manufacturer's protocol. RNA purity and quantification were evaluated using the NanoDrop 2000 spectrophotometer (Thermo Scientific, USA). RNA integrity was assessed using the Agilent 2100 Bioanalyzer (Agilent Technologies, Santa Clara, CA, USA). Then the libraries were constructed using TruSeq Stranded mRNA LT Sample Prep Kit (Illumina, San Diego, CA, USA) according to the manufacturer's instructions.

### Bioinformatics Analysis

The differentially expressed genes (DEGs) were obtained by the cut‐off of │Log2cis‐diaminodichloroplatinumFC│≥ 1, *P* value < 0.05. Then, the DEGs were imported into Database for Annotation, Visualization and Integrated Discovery (DAVID) database (https://david.ncifcrf.gov/) to obtain results of Gene Ontology (GO) and Kyoto Encyclopedia of Genes and Genomes (KEGG) enrichment analyses. The pyroptosis and inflammatory response‐related genes were downloaded from the Gene Set Enrichment Analysis (GSEA) database (https://www.gsea‐msigdb.org/). R software (version 4.2.0) was used to perform GSEA analysis and realize visualization. Proteomic data from the Clinical Proteomic Tumor Analysis Consortium (CPTAC) database were used to analyze the protein expression of GSDME through the University of Alabama at Birmingham Cancer Data Analysis Portal (ULCAN) website (http://ualcan.path.uab.edu)

### Autophagic Flux Measurement

Autophagic flux was detected using adenovirus expressing mCherry‐GFP‐LC3B fusion protein (Ad‐mCherry‐GFP‐LC3B, Cat#C3011, Beyotime Biotechnology). About 5 × 10^4^ cells were seeded in 24‐well plates and infected with Ad‐mCherry‐GFP‐LC3B according to the manufacturer's instructions. Subsequently, cells were treated with drugs and autophagic flux was observed under fluorescence microscopy (Thermo Fisher, EVOS M7000, USA). After the fusion of autophagosome and lysosome, the acidic environment in the lysosome will quench the GFP signals and makes the LC3B fusion protein emit red signals of mCherry. By contrast, when the fusion of autophagosomes and lysosomes was disrupted, the LC3B fusion protein emits yellow signals due to the superposition of GFP and mCherry.

### Annexin V/PI Staining and Cell Cycle Detection

Annexin V‐fluorescein isothiocyanate (FITC)/propidium iodide (PI) apoptosis detection kit (Cat#F6012) and cell cycle detection kit (Cat#C6031) were purchased from Uelandy (Suzhou, China). Cells were treated with drugs for 24 h, after which cell staining was performed according to the manufacturer's instructions. Cell apoptosis and cell cycle were then detected by NovoCyte Flow Cytometer (NovoCyte 2040R, USA)

### Lactate Dehydrogenase Release Assay

Lactate dehydrogenase (LDH) release assay kit (Cat#C0017) was purchased from Beyotime Biotechnology. About 1 × 10^4^ cells were seeded in 96‐well plates. After incubating with drug‐containing culture medium, the LDH release assay was performed according to the manufacturer's instructions.

### Tissue Microarray and Immunohistochemical Staining

The human tissue microarray (TMA) containing 150 ccRCC tissues and 30 normal kidney tissues was purchased from Shanghai OUTDO Biotech Co., Ltd (HKidE180Su02). TMA and the paraffin sections of subcutaneous grafts were deparaffinized, rehydrated, subjected to antigen retrieval, and incubated with specific primary antibodies overnight at 4 °C and secondary antibodies for 1 h at room temperature. Visualization of the immune complexes was achieved using 3,3′‐diaminobenzidine tetrahydrochloride (DAB), followed by hematoxylin counterstaining. The TMA was scanned and imaged using Pannoramic DESK (3D HISTECH, HU). The H‐score was calculated according to the following formula: H‐SCORE = ∑(pi × i) = (percentage of weak intensity × 1) + (percentage of moderate intensity × 2) + (percentage of strong intensity × 3). Human GSDME antibody (Cat#ab230482) was purchased from Abcam (Cambridge, UK). Human Ki‐67 (Cat#9449) and PCNA (Cat#13110) antibodies were purchased from Cell Signaling Technology (Beverly, USA).

### Construction and Transfection of Plasmids and Lentiviruses

The pPLK vector containing small hairpin RNA (shRNA) against human GSDME (pPLK‐shGSDME, Cat#PPL02188‐3a, target sequence: GATGATGGAGTATCTGATCTT) was purchased from Public Protein/Plasmid Library (PPL, Suzhou, China). The Lipofectamine 3000 reagent (#L3000015, Invitrogen, USA) was used to transfect pPLK‐shGSDME and the control plasmid into 786‐O and A‐498 cells according to the manufacturer's instructions.

### Xenograft Model

All animal experiments were conducted in accordance with the animal experimental regulations approved by the Institutional Animal Care and Use Committee of Xiamen University (Ethical approval number: XMULAC20190168). 6–8 week of male immunodeficient nude mice were purchased from the Laboratory Animal Center of Xiamen University and maintained under pathogen‐free conditions. Approximately 5 × 10^6^ A‐498 cells were inoculated subcutaneously into the mice to establish the subcutaneous xenograft model. The mice were randomly divided into four groups and treatment with drugs when the tumor volume reached approximately 30 mm^3^. SGI‐1027 (30 mg kg^−1^ per day) and everolimus (2 mg kg^−1^ per day) were administered once daily by oral gavage. Tumor volume and mice weight were measured every 2 d. The formula for calculating tumor volume was *V* = π (length × width^2^)/6. The formula for calculating tumor growth inhibition (TGI) value was (1‐tumor weight in treatment group/tumor weight in control group) ×100%. After 16 d of treatment, the mice were euthanized and the subcutaneous tumors were isolated, and weighed. The tumors were then divided into two parts, one of which were fixed in formalin for immunohistochemistry staining and the other were stored in liquid nitrogen for immunoblotting.

### Statistical Analyses

All statistical analyses were performed using GraphPad Prism (version 8.0). The IC_50_ values of the drugs were calculated using nonlinear regression. *P*‐value of less than 0.05 was considered the threshold value for statistical significance. The following notations were used: NS (not significant), * (*P* < 0.05), ** (*P* < 0.01), *** (*P* < 0.001) and **** (*P* < 0.0001).

### Ethical Statement

This study was conducted in accordance with the principles of the Declaration of Helsinki and received approval from the Institutional Review Board at Shanghai Outdo Biotech Co., Ltd. (SHYJS‐CP‐1510001).

## Conflict of Interest

The authors declare no conflict of interest.

## Author Contributions

Y.L. and B.G. contributed to this work equally. Y.L., J.X., B.C., and T.W. conceived and designed research; Y.L., B.G., X.D., C.M., A.S., Z.L., D.Y., X.W., Z.S., and Y.W. performed experiments; Y.L., B.G., and T.W. analyzed data. T.W. and Y.L. wrote the manuscript. T.W., P.B. H.H., J.X., and B.C. provided funding. All authors contributed with productive discussions and knowledge to the final version of this manuscript.

## Supporting information

Supporting Information

Supporting Information

## Data Availability

The data that support the findings of this study are available from the corresponding author upon reasonable request.
