# Supplementary material for: Methuosis Inducer SGI‐1027 Cooperates with Everolimus to Promote Apoptosis and Pyroptosis by Triggering Lysosomal Membrane Permeability in Renal Cancer
Source: Adv Sci (Weinh). 2024 Aug 9;11(38):2404693. doi: 10.1002/advs.202404693 (PMC11481186; doi:10.1002/advs.202404693)
Supplement: Supplementary file 1 — Supporting Information [file ADVS-11-2404693-s002.docx]

**Supporting Information**

**Figure S1** Detecting the cytotoxicity of SGI-1027 in HK-2, 786-O, A-498 and Caki-1 cells. HK-2, 786-O, A-498 and Caki-1 cells were treated with SGI-1027 at indicated concentrations for 48 hours.

| A  **** | B  **** |
| --- | --- |

**Figure S2** A, B) 786-O and A-498 cells were treated with 10 μM everolimus, SGI-1027 at indicated concentrations, or their combinations for 24 hours. The cell viability was detected by CCK-8 assay. EVER, everolimus; SGI, SGI-1027. *, *P* < 0.05; ***, *P* < 0.001; ****, *P* < 0.0001.

| A  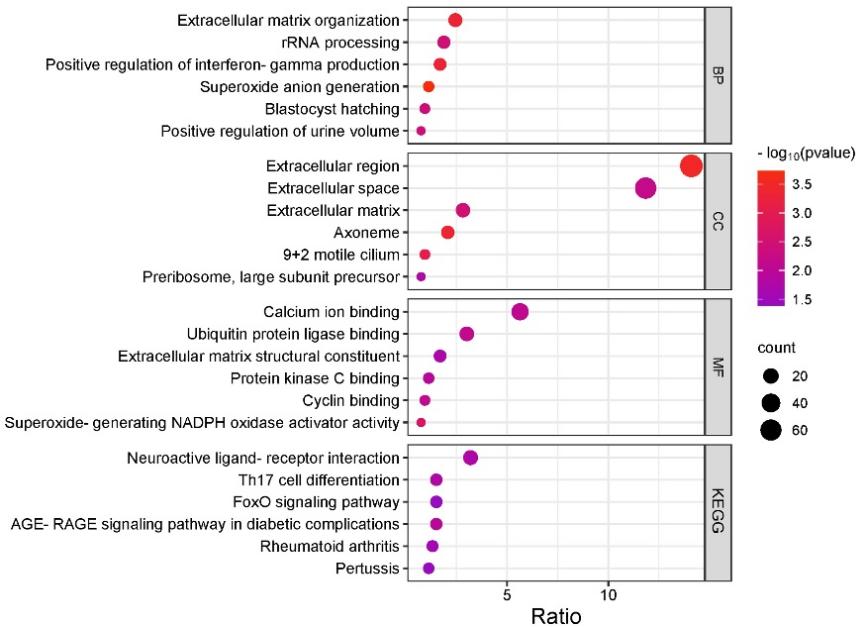 |
| --- |
| B  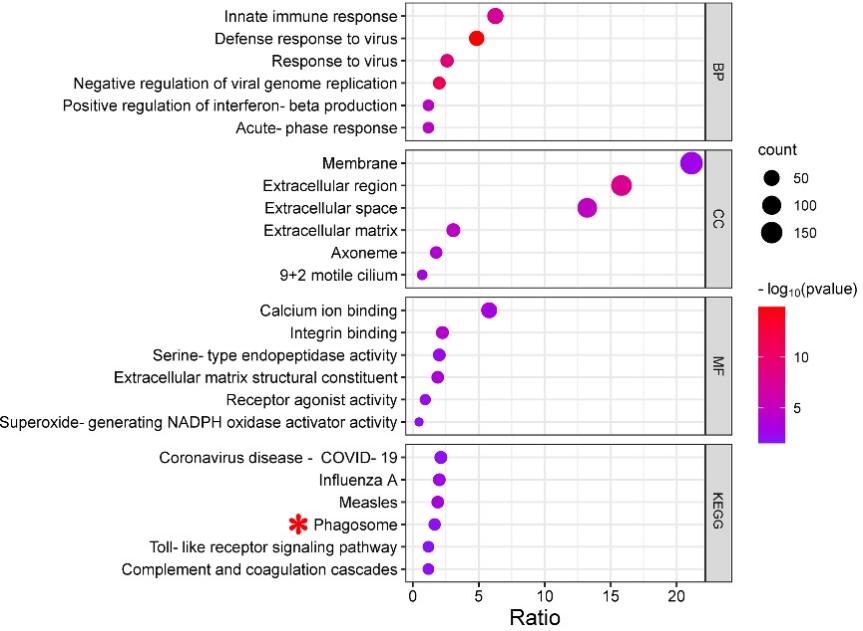 |

| C  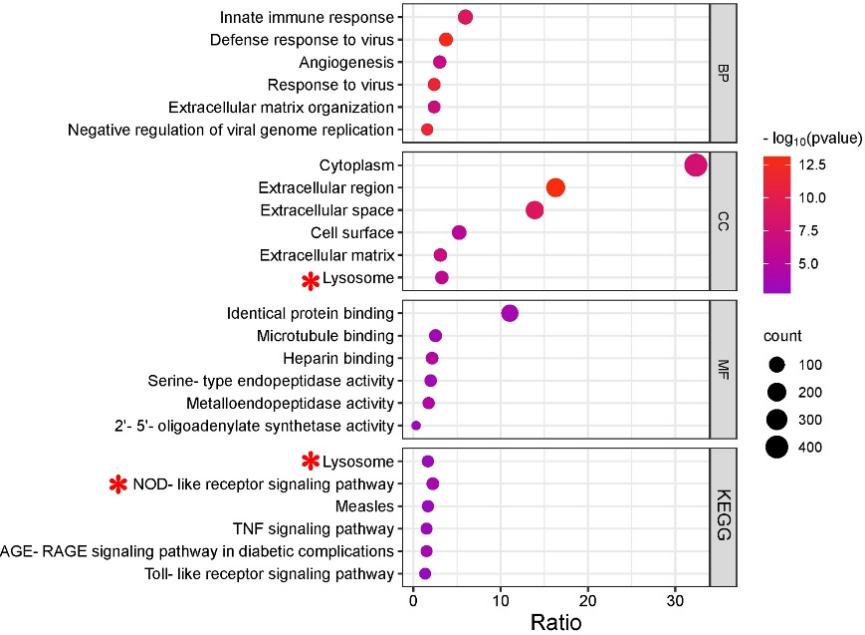 |
| --- |

**Figure S3** A-C) The GO and KEGG enrichment analyses for DEGs in everolimus group A), SGI-1027 group B), combination group C). BP, biological process; CC, cellular component; MF, molecular function; KEGG, kyoto encyclopedia of genes and genomes.

**
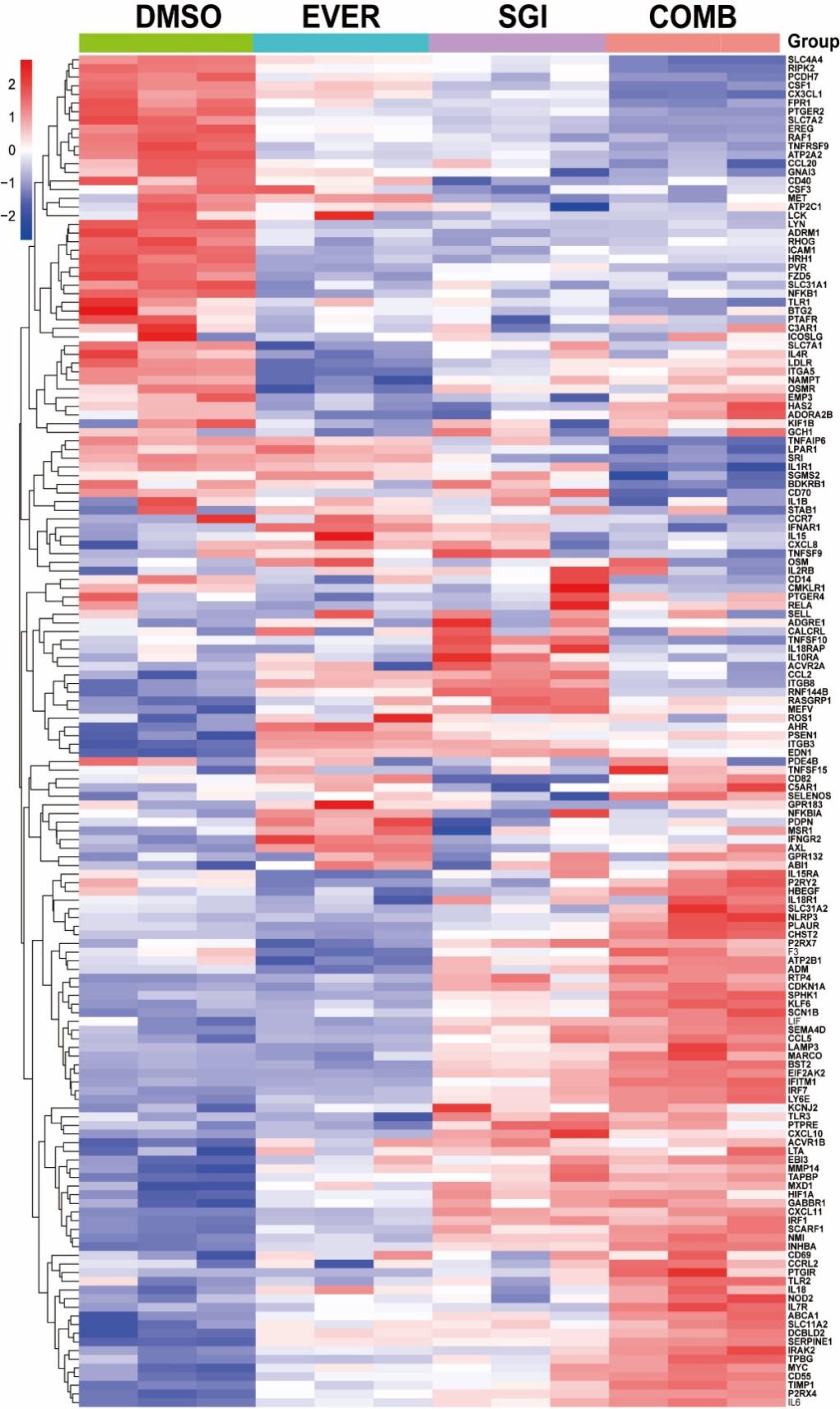
**

**Figure S4** Heatmap of the expression of inflammatory response-related genes in the control group (DMSO), Everolimus (EVER), SGI-1027 (SGI), and combination treatment group (COMB).

| A  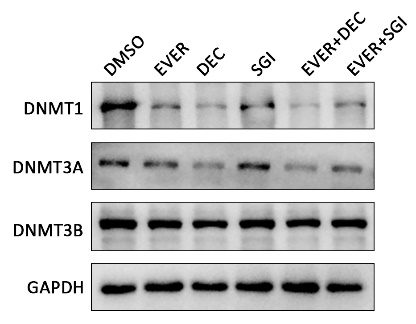 | B  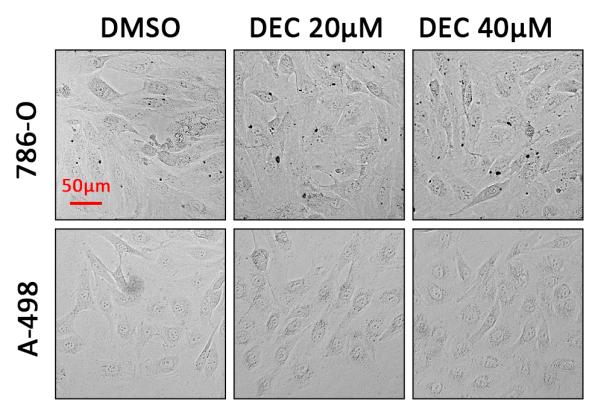 |
| --- | --- |
| C  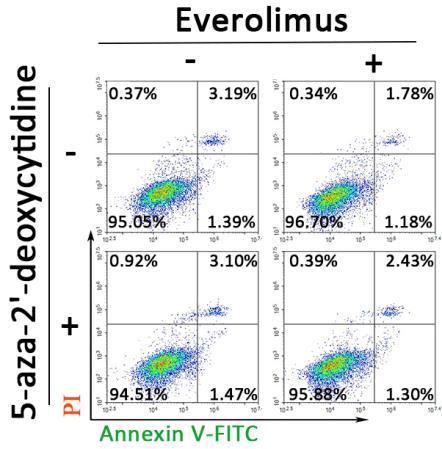 |  |

**Figure S5** A) The effects of 5 µM everolimus (EVER), 10 µM 5-aza-2'-deoxycytidine (DEC), 1.5 µM SGI-1027 (SGI), and their combination on the expression of DNMT family proteins, including DNMT1, DNMT3A, and DNMT3B, were assessed in 786-O cells after 24 hours of treatment. B) 5-aza-2'-deoxycytidine failed to induce vacuolization in 786-O and A-498 cells. 786-O or A-498 cells were subjected to treatment with DMSO, 20 µM, or 40 µM 5-aza-2'-deoxycytidine for 48 hours. Scale bar: 50 µm. C) Annexin V and propidium iodide (PI) staining for 786-O cells treated with 10 µM everolimus, 20 µM 5-aza-2'-deoxycytidine, or their combination for 48 hours.

| A  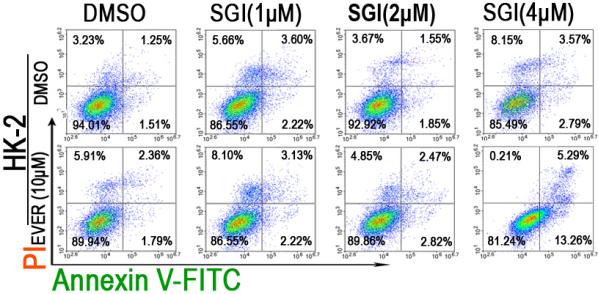 |  |
| --- | --- |
| B  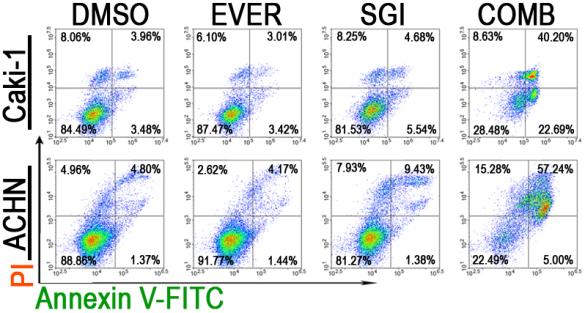 |   |
| C | |
| 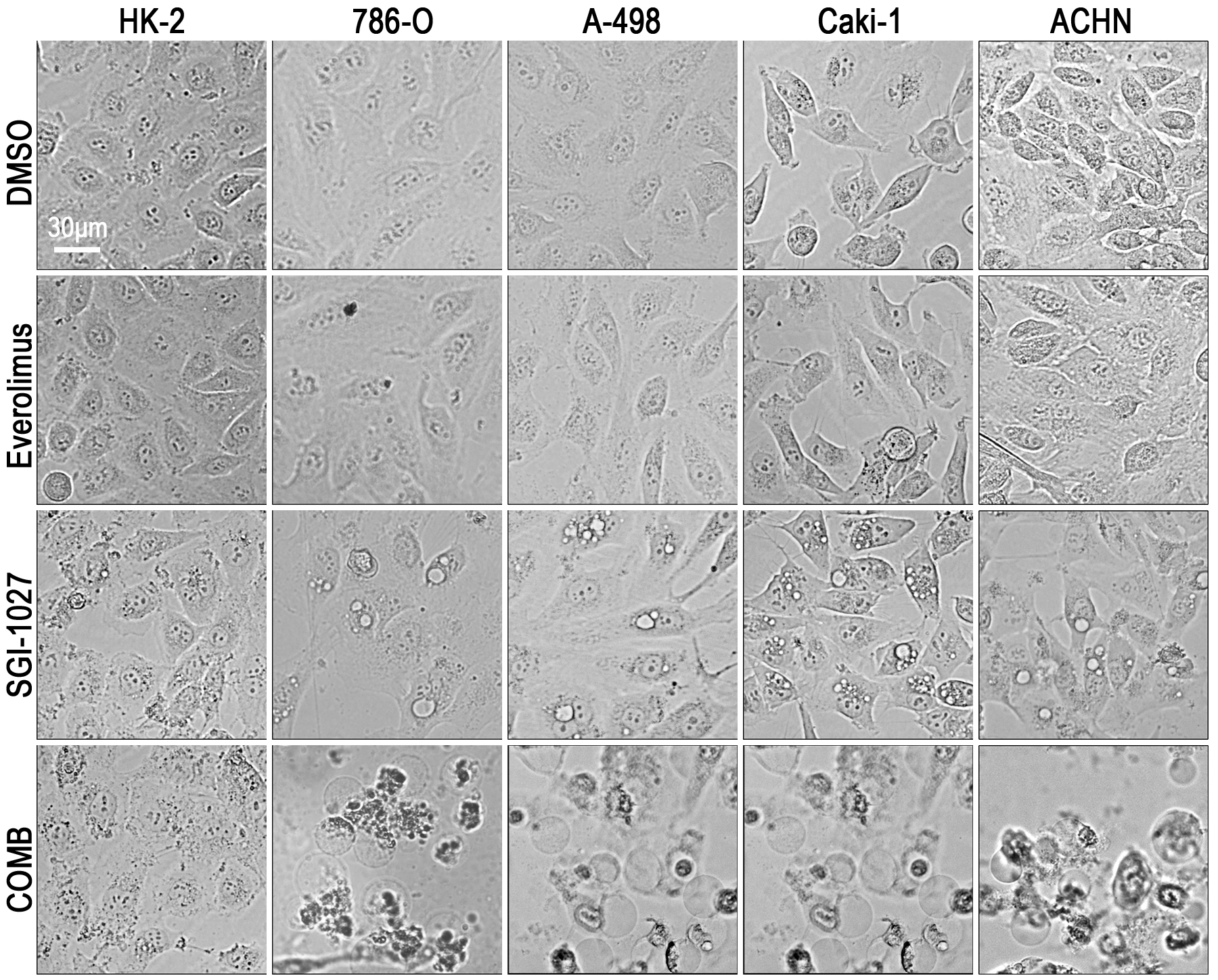 | |

**Figure S6** A) Annexin V and propidium iodide (PI) staining for HK-2 cells treated with 10 μM everolimus, different concentration of SGI-1027 or their combination as indicated for 24 hours. B) Annexin V and propidium iodide staining for Caki-1 and ACHN cells treated with 10 μM everolimus, 4 μM SGI-1027 or their combination for 24 hours. C) Optical microscopy images of HK-2, 786-O, A-498, Caki-1, ACHN cells treated with DMSO (control), 10 μM everolimus, 4 μM SGI-1027, or their combination for 24 hours. EVER, everolimus; SGI, SGI-1027; COMB, SGI-1027 combined with everolimus. ***, *P* < 0.001; ****, *P* < 0.0001.

| A   | B   | C   |
| --- | --- | --- |
| D  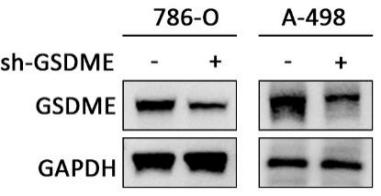 | E   | F   |

**Figure S7** A-C) In the presence of 5 µM everolimus, the IC50 of SGI-1027 was determined in HK-2 cells (A), 786-O cells (B), and A-498 cells (C). MOS (Margin of Safety) represents the ratio of the IC50 value in HK-2 cells to the IC50 values in 786-O and A-498 cells. D) GSDME was knockdown in 786-O and A-498 cells. E, F) Knockdown of GSDME reduced the cytotoxicity of SGI-1027 combined with everolimus. The changes in viability of 786-O and A-498 cells were assessed after GSDME knockdown, treatment with a combination of 1.5 µM SGI-1027 and 5 µM everolimus, or both. **, *P* < 0.01.
